# Insulin-independent stimulation of skeletal muscle glucose uptake by low-dose abscisic acid via AMPK activation

**DOI:** 10.1038/s41598-020-58206-0

**Published:** 2020-01-29

**Authors:** Mirko Magnone, Laura Emionite, Lucrezia Guida, Tiziana Vigliarolo, Laura Sturla, Sonia Spinelli, Ambra Buschiazzo, Cecilia Marini, Gianmario Sambuceti, Antonio De Flora, Anna Maria Orengo, Vanessa Cossu, Sara Ferrando, Ottavia Barbieri, Elena Zocchi

**Affiliations:** 1 Department of Experimental Medicine, Section of Biochemistry, University of Genova, Viale Benedetto XV, 1, 16132 Genova, Italy; 2 Nutravis S.r.l., Via Corsica 2/19, 16128 Genova, Italy; 3 Animal Facility, IRCCS Ospedale Policlinico San Martino, Largo Benzi 10, 16132 Genova, Italy; 4 Nuclear Medicine, IRCCS Ospedale Policlinico San Martino, Largo Benzi 10, 16132 Genova, Italy; 5 CNR Institute of Bioimages and Molecular Physiology, Milan, Italy; 6 Department of Health Sciences, Via A. Pastore 1, Genova, Italy; 7 Department of Earth, Environmental and Life Sciences, University of Genova, Corso Europa 26, Genova, Italy

**Keywords:** Biochemistry, Endocrinology

## Abstract

Abscisic acid (ABA) is a plant hormone active also in mammals where it regulates, at nanomolar concentrations, blood glucose homeostasis. Here we investigated the mechanism through which low-dose ABA controls glycemia and glucose fate. ABA stimulated uptake of the fluorescent glucose analog 2-NBDG by L6, and of [^18^F]-deoxy-glucose (FDG) by mouse skeletal muscle, in the absence of insulin, and both effects were abrogated by the specific AMPK inhibitor dorsomorphin. In L6, incubation with ABA increased phosphorylation of AMPK and upregulated PGC-1α expression. LANCL2 silencing reduced all these ABA-induced effects. *In vivo*, low-dose oral ABA stimulated glucose uptake and storage in the skeletal muscle of rats undergoing an oral glucose load, as detected by micro-PET. Chronic treatment with ABA significantly improved the AUC of glycemia and muscle glycogen content in CD1 mice exposed to a high-glucose diet. Finally, both acute and chronic ABA treatment of hypoinsulinemic TRPM2^-/-^ mice ameliorated the glycemia profile and increased muscle glycogen storage. Altogether, these results suggest that low-dose oral ABA might be beneficial for pre-diabetic and diabetic subjects by increasing insulin-independent skeletal muscle glucose disposal through an AMPK-mediated mechanism.

## Introduction

Abscisic acid is an isoprenoid hormone which plays important roles in the regulation of plant responses to environmental stress. ABA is also present and active in lower Metazoa (Porifera and Hydroids), where it regulates the sponge response to an increase in water temperature and light-induced tissue regeneration in hydroids^1,2^. ABA is present as an endogenous hormone also in humans^3^, where it regulates innate immune cell function^4,5^, the expansion of hemopoietic progenitors^6^ and glucose homeostasis^3,7^. Conservation of ABA across the plant and animal kingdoms points to its very early evolution, in a common precursor to Metaphyta and Metazoa, as a messenger involved in the physiological adaptation of cells and organisms to changing environmental conditions. This general role of ABA in the living is in accordance with its most recently unveiled role in the regulation of blood glucose levels^7^.

The response to hyperglycemia in mammals is mainly controlled by the interplay between two peptide hormones: insulin, released by pancreatic islet β-cells stimulated by high extracellular glucose levels, and the incretin glucagon-like peptide 1 (GLP-1), released by entero-endocrine cells stimulated by nutrients in the gut. GLP-1 in turn stimulates insulin release and suppresses the release of glucagon, the main glycemia-increasing hormone, from pancreatic islet α-cells.

Several lines of evidence indicate that ABA is a new and important player in mammalian glycemic control. Nanomolar ABA stimulates insulin-independent glucose uptake by murine adipocytes *in vitro* by increasing the expression and plasmamembrane translocation of the glucose transporter GLUT4, also the target of insulin^3,8^. Plasma ABA increases after an oral glucose load in healthy humans, but not in subjects with type 2 diabetes (T2D), or with gestational diabetes (GDM). In the latter case, the resolution of the diabetic state that follows childbirth is accompanied by the restoration of a normal ABA response to oral glucose^9^. ABA stimulates the glucose-independent release of GLP-1 from enteroendocrine cells *in vitro* and the increase of plasma GLP-1 in fasted rats^10^. Finally, low-dose oral ABA reduces glycemia and also insulinemia in rats and in healthy humans undergoing a glucose load^7^.

The fact that ABA administration reduces both glycemia and insulinemia suggested that the mechanism underlying the glycemia-lowering action of low-dose ABA *in vivo* could depend on the stimulation of peripheral glucose uptake^7^. The identification of a second hormone beside insulin capable of stimulating muscle glucose uptake would bear significant consequences in clinical conditions where insulin deficiency or insulin resistance reduce glucose tolerance.

The aim of this study was, (i) to explore the effect of ABA in the absence of insulin on myocyte glucose uptake *in vitro* and *ex vivo*; (ii) to investigate the effect of a single low-dose ABA administration on muscle glucose disposal *in vivo* by micro-PET, and (iii) to verify whether low-dose ABA improves glucose tolerance in hypoinsulinemic mice.

## Results

### ABA stimulates glucose uptake in the absence of insulin via an AMPK-dependent mechanism

**Figure 1:**
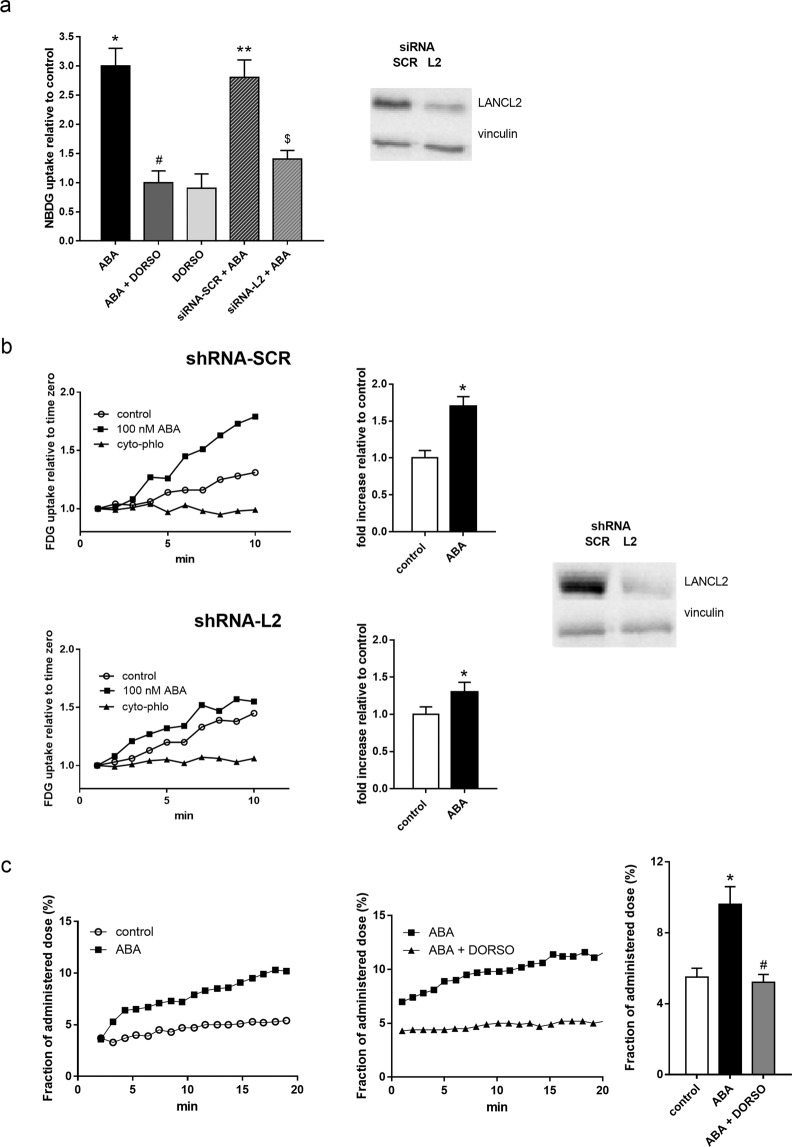
Glucose uptake in rat L6 myoblasts and in murine muscle incubated with ABA: effect of AMPK inhibition and LANCL2 silencing. (**a**) Serum-starved rat L6 myoblasts were: (i) pre-incubated for 30 min without (control) or with the AMPK inhibitor dorsomorphin (1 µM), or (ii) transiently transfected with scramble (siRNA-SCR) or LANCL2-targeting siRNA (siRNA-L2), then incubated without (control) or with 100 nM ABA, without or with 1 µM dorsomorphin, for 30 min and uptake of the fluorescent glucose analog 2-NBDG was measured after 10 min incubation with the dye. Results are expressed as fluorescence relative to control (mean ± SD from at least 3 experiments; *p = 0.001 relative to control, ^#^p = 0.002 relative to ABA, **p = 0.001 relative to siRNA-SCR without ABA, ^$^p = 0.002 relative to siRNA-SCR + ABA). Inset: a representative Western blot of LANCL2 expression in siRNA-SCR vs. siRNA-L2 cells. (**b**) Ligand Tracer analysis of FDG uptake by L6 cells stably infected with a scramble (shRNA-SCR, upper panel) or with a LANCL2-targeting shRNA (shRNA-L2, lower panel). Cells were pre-incubated with or without 100 nM ABA for 1 hour before being placed in the Ligand Tracer device. Cytochalasin B and Phloretin were added at time zero of the Ligand Tracer analysis (cyto-phlo). Representative traces are shown on the left and mean ± SD values from 3 experiments are shown on the right. Inset: a representative Western blot of LANCL2 expression in shRNA-SCR vs. shRNA-L2 cells. *p < 0.03 relative to control. (**c**) Freshly isolated samples of femoral quadriceps (~100 mg) were pre-incubated, or not (control) with 1 microM dorsomorphin (Dorso) and then time-activity curves of FDG uptake were recorded, in the absence (control) and in the presence of 100 nM ABA. Representative curves are shown on the left and central panel (without and with Dorso, respectively) and the mean ± SD from 3 experiments is shown on the right panel. *p = 0.007 relative to control, #p = 0.007 relative to ABA.

Previous studies had shown that ABA stimulated glucose uptake by murine 3T3-L1 preadipocytes in the absence of insulin, by increasing the expression and the plasmamembrane translocation of the insulin-sensitive glucose transporter GLUT4 via a PI3K/Akt-dependent pathway^3,8^. In skeletal muscle, GLUT4 translocation to the plasmamembrane and glucose transport are known to be stimulated by AMPK^11^. Expression of the ABA receptor LANCL2 in L6 was preliminarily confirmed by Western blot, which also showed presence of closely related LANCL1, though at lower levels, as confirmed by RT-PCR (not shown). The effect of ABA on glucose transport was thus explored on rat L6 myoblasts, in the absence or presence of the AMPK inhibitor dorsomorphin. Nanomolar ABA stimulated uptake of the fluorescent glucose analog 2-NBDG in serum-starved L6 myoblasts, confirming previous results obtained with radioactive glucose^3^; this effect was abrogated when cells were preincubated with 1 µM dorsomorphin (Fig. 1a, light grey bar). Addition of 100 nM insulin stimulated NBDG uptake in serum-starved L6 cells, quantitatively similarly to 100 nM ABA (approx. 3-fold) and pre-incubation of the cells for 30 min with 100 nM wortmannin, a specific PI3K inhibitor, reduced NBDG uptake to values similar to those measured in untreated control cells, similarly to what observed in ABA-treated cells pre-incubated with dorsomorphin (not shown). Thus, the mechanism through which ABA stimulates NBDG uptake in L6 is AMPK-dependent and different from the one of insulin. ABA-stimulated NBDG uptake was significantly reduced by silencing of LANCL2 with specific siRNAs (Fig. 1a, grey striped bar). siRNA-LANCL2-transfected L6 showed a reduction of approximately 80% of mRNA and 70% of protein levels compared with control cells, transfected with the siRNA-SCR (inset to Fig. 1a).

In order to obtain a higher extent of LANCL2 silencing and to explore the time-course of glucose uptake, a different experimental approach was undertaken. L6 cells were infected with lentiviral particles expressing either shRNA-SCR (control cells) or shRNA-L2 (shRNA-L2 cells). After puromycin selection, shRNA-L2 cells showed a > 95% reduction of LANCL2 mRNA levels by Real Time PCR and a > 90% reduction of protein levels (by Western blot) compared with control cells (inset to Fig. 1b). Using the Ligand Tracer device, [^18^F]-deoxy-glucose (FDG) uptake in shRNA-L2 L6 cells was measured in real-time and compared with that of control cells, shRNA-SCR, incubated with or without ABA. ABA increased FDG uptake in shRNA-SCR cells approximately 1.7-fold compared with untreated cells (Fig. 1b, upper panel) and LANCL2 silencing reduced the ABA-induced stimulation of FDG uptake (Fig. 1b, lower panel). In the presence of cytochalasin B and phloretin, FDG uptake was similarly abrogated in control (cyto-phlo Fig. 1b), as well as in ABA-treated cells (not shown), indicating that the FDG uptake measured was indeed the result of glucose transport. Finally, a 10-minute pre-incubation of shRNA-SCR cells with 0.2 mM Indinavir (a GLUT4-specific inhibitor of glucose transport) reduced the effect of ABA on FDG uptake in L6 cells by 93 ± 5%, confirming that stimulation by ABA of glucose transport occurs via GLUT4 (not shown).

To confirm the insulin-independent stimulation by ABA of glucose transport in skeletal muscle *ex vivo* quadriceps samples (~100 mg) were isolated from C57BL6 mice after overnight starvation to directly measure muscle uptake of FDG using the Ligand Tracer tool. Pre-incubation of the tissue for 30 min with 100 nM ABA resulted in an approximately 2-fold increase of FDG uptake, and eventually of retained dose, compared with control samples, pre-incubated without the hormone, as observed over a time span of approximately 20 min (Fig. 1c, left panel). Pre-incubation of muscle samples with ABA in the presence of dorsomorphin abrogated the ABA-induced stimulation and restored a kinetics of FDG uptake similar to that of ABA-untreated muscle (Fig. 1c, central panel). Indeed, mean FDG uptake (expressed as % of administered activity per gram of tissue) was reduced to control value by the AMPK inhibitor (Fig. 1c, right panel).

Altogether, results obtained *in vitro* on L6 myoblasts and *ex vivo* on mouse muscle biopsies indicated that ABA stimulated glucose uptake in the absence of insulin and that the effect was mediated at least in part by the ABA receptor LANCL2, and by activation of AMPK.

### ABA increases AMPK phosphorylation and upregulates PGC-1α expression

**Figure 2:**
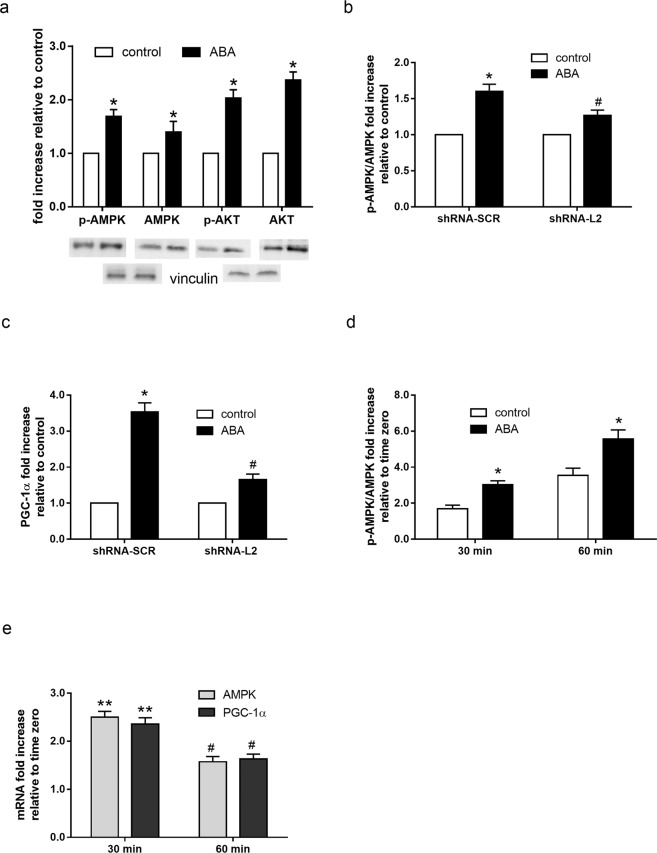
Effect of ABA on the p-AMPK/AMPK ratio and on AMPK, Akt and PGC1α expression levels in L6 cells and in murine muscle. (**a**) Serum-starved L6 myoblasts were incubated without (white bars) or with 100 nM ABA (black bars) for 60 min. pAMPK (Thr172) and pAkt (Ser473) and the respective total proteins were analyzed by Western blot. The mean ± SD from at least 3 experiments is shown. *p < 0.03 relative to control. (**b,c**) L6 cells stably infected with a scramble (shRNA-SCR) or with a LANCL2-targeting shRNA (shRNA-L2) were incubated without (control) or with 100 nM ABA for 60 min. (**b**) Western blot analysis of the pAMPK (Thr172)/AMPK ratio. (**c**) RT-PCR analysis of PGC-1α mRNA. The mean ± SD from 3 experiments is shown. *p < 0.01 relative to control, ^#^p < 0.02 relative to control. (**d,e**) Small samples of femoral quadriceps were incubated without (control) or with 100 nM ABA for 30 or 60 min; the pAMPK/AMPK ratio was analyzed by Western blot (**d**) and the mRNA levels of AMPK and of PGC-1α were determined by RT-PCR (**e**). The mean ± SD from 3 experiments is shown. *p < 0.01 relative to control; **p < 0.01 and ^#^p < 0.03 relative to the respective time zero.

Phosphorylation of AMPK at Thr172 increased in L6 cells incubated with ABA, compared with untreated controls (Fig. 2a). Besides inducing its phosphorylation, ABA also increased total AMPK levels by approximately 40% compared with untreated L6 cells (Fig. 2a); the increase of total AMPK, which required the use of vinculin as reference protein instead of total AMPK (see Methods), was confirmed by RT-PCR, which showed a 2-fold increase of AMPK mRNA in L6 treated with 100 nM ABA for 60 min compared with controls (n = 3; p = 0.003). The similar extent of increase induced by ABA on AMPK protein levels and on its phosphorylation resulted in a pAMPK/AMPK ratio of ~1. Downstream of activated AMPK an increased Ser phosphorylation of the Rab GTPase activating protein TBC1D1, which regulates GLUT4 plasmamembrane translocation, was observed in the presence of 100 nM ABA: the ratio pTBC1D1/total TBC1D1 increased 2.5-fold relative to untreated L6. ABA also induced an approximately 2-fold increase of pAkt (Ser473) and of total Akt in L6 (Fig. 2a), again resulting in a ratio of phosphorylated/total protein of ~1.

Interestingly, ABA treatment also induced an approximately 2-fold increase of LANCL2 protein expression, as detected by Western blot (1.8 ± 0.2, p < 0.05, n = 3).

AMPK phosphorylation was investigated by Western Blot also on ABA-treated shRNA-SCR and shRNA-L2 cells. In shRNA-SCR cells, ABA induced an increase of the p-AMPK/AMPK (1.6 ± 0.1 fold compared with untreated cells, Fig. 2b); conversely, a reduced effect of ABA was observed in shRNA-L2 cells (p-AMPK/AMPK ratio of 1.3 ± 0.1), suggesting a role for LANCL2 in the modulation of AMPK phosphorylation induced by ABA. The transcription factor PGC-1α is among the target proteins of AMPK in skeletal muscle and in turn regulates transcription of several genes including GLUT4^12,13^. The expression of PGC-1α in shRNA-SCR and shRNA-L2 cells was analyzed by Real Time PCR: ABA upregulated the expression of PGC-1α (3.5 ± 0.2 fold relative to control cells, Fig. 2c) and this effect was reduced (1.7 ± 0.1 fold) in shRNA-L2 cells.

Finally, the pAMPK/AMPK ratio and the expression levels of AMPK and of PGC-1α were investigated in *ex-vivo* experiments on murine quadriceps muscle samples, incubated without (control) or with 100 nM ABA. The pAMPK/AMPK ratio increased approximately 3- and 5-fold at 30 and 60 min incubation, respectively, in ABA-treated compared with control muscles (Fig. 2d). At the same time points, the expression of both AMPK and PGC-1α was also upregulated in the ABA-treated muscles, approximately 2.5- and 1.5-fold relative to untreated controls (Fig. 2e).

### Low-dose oral ABA increases blood glucose clearance and skeletal muscle uptake in rats

Male, 7-week old Wistar rats received an oral glucose load (OGTT) of 1 g glucose/Kg body weight (BW) with or without (controls) ABA (1 µg/Kg BW), administered together with the glucose solution. Immediately after gavage, the animals were anesthetized, placed on the bed of a micro-PET scanner and 30–45 MBq of FDG was injected into the tail vein.

**Figure 3:**
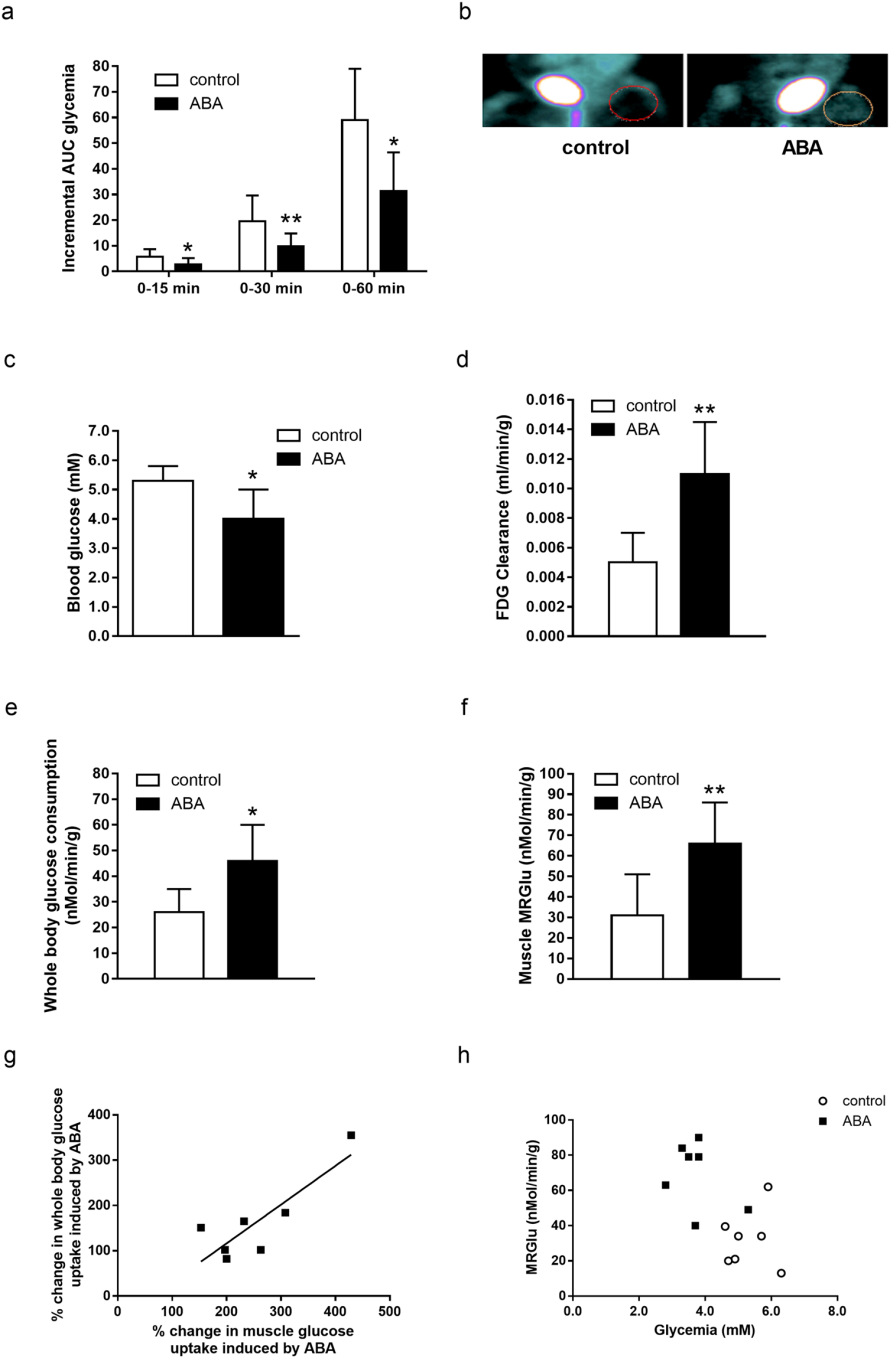
Micro-PET analysis of FDG disposal in ABA-treated and control rats during OGTT. Fasted male Wistar rats (7/group) were subjected to an OGTT without (control) or with ABA at 1 µg/Kg BW (ABA) and micro-PET images were acquired, as detailed in the Methods section. (**a**) AUC of glycemia over the indicated time frames. (**b**) An example of MRGlu images obtained by dynamic micro-PET; (**c**) blood glucose levels at tracer injection; (**d**) blood FDG clearance; (**e**) whole-body glucose consumption; (**f**) skeletal muscle glucose consumption (MRGlu); (**g**) percentage change of whole body glucose uptake relative to muscle glucose consumption in the ABA-treated animals (Pearson; p = 0.015); (**h**) muscle MRGlu relative to glycemia at tracer injection (Pearson; p = 0.014). *p ≤ 0.02; **p ≤ 0.003.

ABA-treated rats showed a significantly reduced glycemia profile and consequently a lower plasma glucose AUC (Fig. 3a) compared with controls, in line with previous results^7^. The AUC of plasma insulin in the ABA-treated rats was approximately ten times lower compared with controls (not shown), in agreement with previously published results^7^.

Images obtained by dynamic micro-PET during the OGTT (two representative examples are shown in Fig. 3b) were analyzed to calculate blood FDG clearance and tissue FDG uptake. At tracer injection, blood glucose levels were significantly lower in ABA-treated compared with control rats (4.0 ± 1.0 vs. 5.3 ± 0.7 mM, respectively, p = 0.013; Fig. 3c). In agreement with this observation, ABA markedly increased blood FDG clearance, from 4.98 ± 1.81 µL × min^−1^ × g^−1^ BW in controls to 11.10 ± 3.59 µL × min^−1^ × g^−1^ BW in ABA-treated rats (p = 0.0013; Fig. 3d) and overall whole-body (WB) glucose consumption, which increased approximately 2-fold from 26.1 ± 8.5 nmol × min^−1^ × g^−1^ BW to 46.3 ± 14.3 nmol × min^−1^ × g^−1^ BW, in control vs. ABA-treated rats, respectively (p = 0.016; Fig. 3e). The increase in WB glucose consumption induced by ABA quantitatively agreed with the higher glucose disposal measured in skeletal muscles, where MRGlu was increased 2-fold in the ABA-treated animals compared with controls (from 32.0 ± 16.0 nmol min^−1^ × g^−1^ BW to 66.0 ± 20.0 in control and ABA-treated rats, respectively; p = 0.003; Fig. 3f), similarly to what observed in the *ex vivo* experiments (Fig. 1c). Indeed, the percentage change in glucose disposal in muscle and WB in the ABA-treated vs. –untreated rats showed a linear correlation (Pearson’s r = 0.85; p = 0.015; Fig. 3g), suggesting that WB glucose consumption was largely dependent upon the response of skeletal muscle to ABA. When muscle MRGlu was plotted against glycemia, values from the OGTTs with ABA were separated from those obtained in the experiment without ABA, but both sets of data showed an inverse correlation with glycemia (Pearson’s r = −0.64; p = 0.014; Fig. 3h), indicating that the higher the muscle capacity for glucose disposal, the lower the glycemia values.

The interscapular location of brown adipose tissue (BAT) in rodents also allowed acquisition of the BAT-specific signals, revealing a similar 2-fold increase of FDG uptake also in the BAT of ABA-treated *vs*. control animals, as already reported^8^.

Altogether, results from the micro-PET experiments confirmed a faster decrease of plasma glucose levels in the ABA-treated animals, in line with the decreased AUC of plasma glucose, and an increased glucose uptake by skeletal muscle.

### Increased physical performance, skeletal muscle glycogen content and improved glucose tolerance in chronically ABA-treated mice

Stimulation of skeletal muscle glucose uptake by low-dose oral ABA in rats prompted us to investigate whether chronic ABA treatment would ameliorate glucose tolerance and induce a measurable effect on skeletal muscle performance and glycogen content. To this end, male CD1 mice (8/group) were fed a high-glucose diet (1 g glucose/Kg BW), with or without (control) ABA at 1 µg/Kg BW, both glucose and ABA being administered with the drinking water. After 4 weeks, fasting blood glucose (FBG) of the ABA-treated mice was not significantly different compared with that of the untreated animals (3.94 ± 0.22 vs. 4.05 ± 0.61 mM, respectively). Likewise, the mean BW of the ABA-treated animals was not significantly different compared with that of untreated controls.

**Figure 4:**
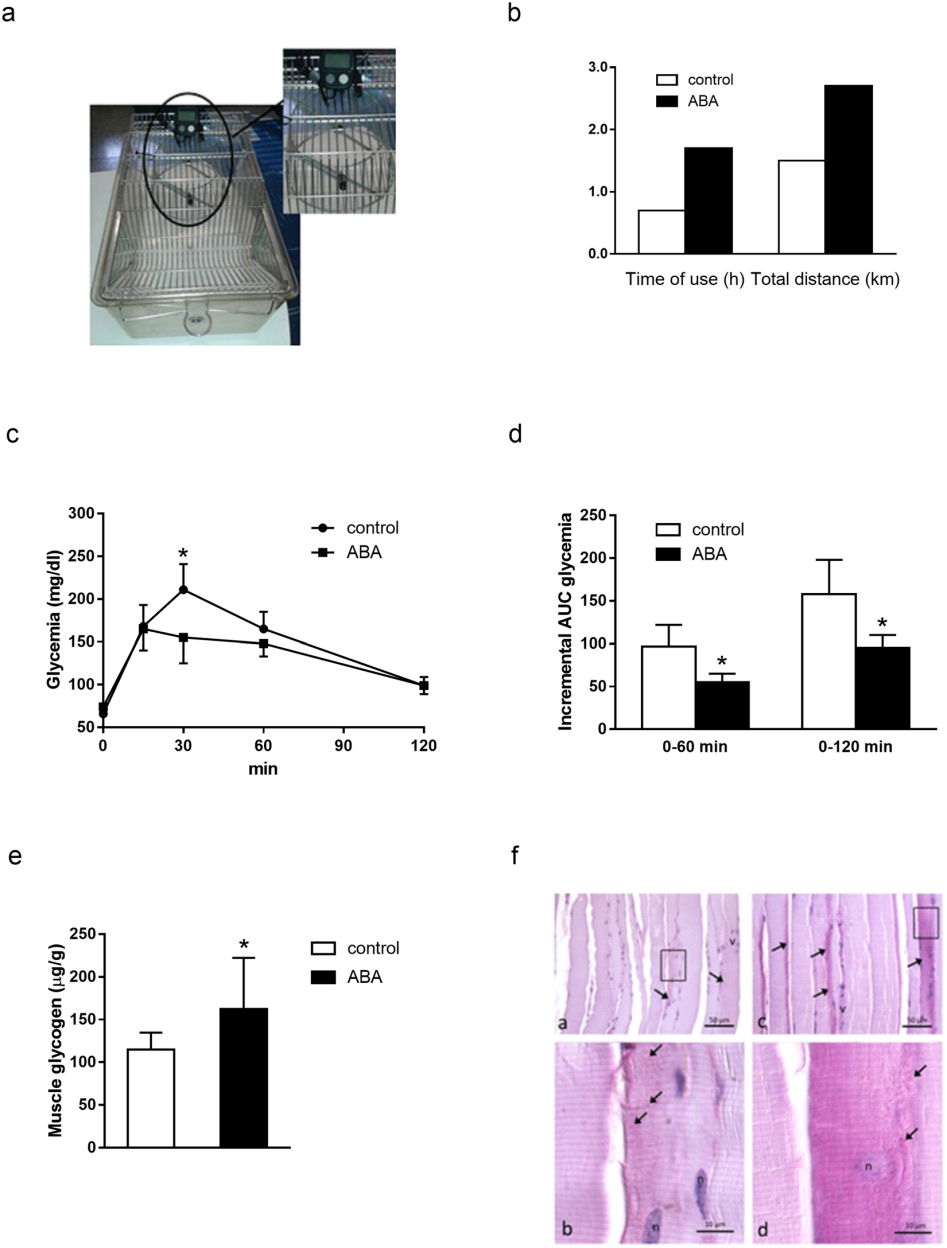
Physical performance, OGTT and skeletal muscle glycogen content in ABA-treated mice fed a high-glucose diet. Male CD1 mice (8/group) were administered glucose with the drinking water without (control) or with the addition of ABA at a concentration calculated to provide a daily dose of ABA of approximately 1 µg/Kg BW (ABA). (**a,b**) After 4 weeks, control and ABA-treated mice were transferred for 12 hours in separate cages, each equipped with a wheel connected to a digital speedometer (**a**), measuring the total distance traveled on the wheel and the time of rotation of the wheel. The tests were performed during the night time on animals with freely available food and water. (**c**) glycemic profile and (**d**) AUC of glycemia during OGTT performed after 5 weeks; (**e,f**) glycogen content in skeletal muscle after 5 weeks, as determined by biochemical analysis on muscle lysates (**e**) and histochemical analysis of muscle sections (**f**): panels a and c, low magnification images, panels b and d, high magnifications of the framed areas in panels a and c, respectively. Arrows indicate glycogen accumulation. *p ≤ 0.02.

At the end of the 4^th^ week of treatment male mice underwent a standardized test to compare the physical performance of the ABA-treated and of the untreated animals. To this end, a running wheel connected to a digital speedometer (Fig. 4a) recording the time of use and the total distance travelled was placed in each cage and the parameters were recorded for 12 hours during night time. There was no coercion to use the wheel; thus, the test measured the spontaneous physical activity of the animals in the absence of preliminary training. The ABA-treated animals showed an appreciable improvement of muscular performance: ABA-treated mice kept the wheel rotating for a longer time (~2.5-fold) compared with controls, travelled a significantly longer total distance (~2-fold) at the end of the 12-hours recording time (Fig. 4b), and recorded a maximal speed 3.2 times higher compared with that of the controls.

Three days after the wheel test, the same animals underwent an OGTT, to evaluate their glucose tolerance. ABA-treated mice showed a significantly reduced glycemic profile (Fig. 4c) and, consequently, a significantly reduced glycemia AUC after glucose load compared with untreated controls: the incremental AUC of glycemia was 94.7 ± 16.3 vs. 158 ± 39.7 in ABA-treated vs. control animals, respectively (p = 0.011; Fig. 4d).

Finally, 4 days after the OGTT, mice were fasted for 4 hours before sacrifice, to reduce inter-individual variations related to feeding activity, and the glycogen content in the hind limb muscles was analyzed. Glycogen content was found to be significantly higher in the ABA-treated animals compared with the controls, both by enzymatic assay (Fig. 4e) and by histological staining (Fig. 4f). An increased glycogen content in skeletal muscle, without any increase in hepatic glycogen, has been shown to improve physical endurance in mice^14^.

Altogether, these results indicate that a 5-week long, low-dose ABA administration in a high-glucose diet ameliorates glucose tolerance and increases skeletal muscle glycogen content and physical endurance in mice.

### ABA improves glucose tolerance and increases muscle glycogen in hypoinsulinemic TRPM2-KO mice

**Figure 5:**
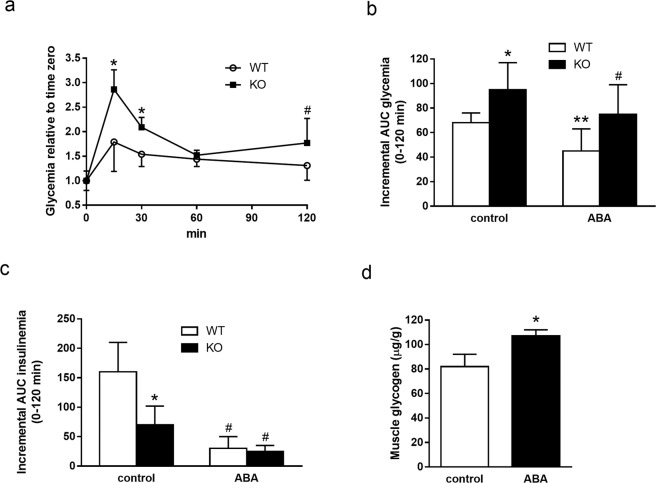
OGTT and skeletal muscle glycogen content in ABA-treated TRPM2 knock-out mice. Male C57 BL/6 mice (6/group), wild type (WT) or knock-out for TRPM2 (KO) underwent an OGTT without (control) or with ABA at 1 μg/Kg BW (ABA): (**a**) glycemia profile *p ≤ 0.006, ^#^p = 0.027, KO vs. WT; (**b**) incremental AUC of glycemia; *p = 0.023, KO vs. WT; **p = 0.024, ABA vs. control in WT; ^#^p = 0.044 ABA vs. control in KO; (**c**) incremental AUC of insulinemia; *p = 0.001 WT vs. KO; ^#^p < 0.001 ABA vs. control in WT and in KO; (**d**) skeletal muscle glycogen content in TRPM2 KO mice after 5 weeks treatment with glucose in the drinking water, without (control) or with the addition of 1 μg/Kg BW ABA (ABA). *p = 0.022 ABA vs. control.

TRPM2-KO mice were previously described to be affected by impaired insulin secretion under conditions of glucose load^15^. OGTTs performed in TRPM2-KO mice confirmed these results, showing a significantly higher glycemia profile (Fig. 5a) and AUC of glycemia (Fig. 5b), and a significant reduction of insulin secretion (Fig. 5c) in KO (black bars) compared with wild-type (WT) mice (white bars). When the OGTTs were performed with 1 μg/Kg BW ABA, the AUC of glycemia was significantly reduced not only in the WT mice, as expected from previous results (Fig. 3a), but also in the TRPM2-KO mice (Fig. 5b). Notably, the improvement of the AUC of glycemia was associated with a reduced AUC of insulinemia both in WT and in TRPM2-KO mice, as compared with the respective OGTTs performed in the absence of ABA (Fig. 5c), indicating an insulin-independent ability of ABA to improve glycemic control. Finally, the daily treatment of TRPM2-KO mice for 5 weeks with a high-glucose diet (1 g glucose/Kg BW, administered in the drinking water) containing ABA at 1 µg/Kg BW increased muscle glycogen content compared with TRPM2-KO controls, fed the same high-glucose diet without ABA (Fig. 5d), similarly to what previously observed in normoinsulinemic CD1 mice (Fig. 4e). These results suggest that ABA supplementation can ameliorate glucose tolerance and improve muscle glucose uptake under conditions of defective insulin release.

## Discussion

The hypoglycemic action of insulin largely depends on its ability to stimulate glucose uptake and metabolism by adipose tissue and skeletal muscle, the latter accounting for approximately 70–80% of the total body glucose consumption^16^. Insulin alone is capable of this action; thus, the unique role of this hormone in the regulation of peripheral glucose uptake during hyperglycemia represents a bottleneck in the physiology of whole body glucose disposal and in the pharmacological approaches to improve its dysregulation. As Nature always provides multiple mechanisms for physiological functions that are essential to cell and species survival, the identification of a new hormone with the capacity to stimulate muscle glucose uptake independently of insulin fulfils a general principle of redundancy and is amenable to clinical exploitation.

Abscisic acid (ABA) is an isoprenoid plant stress hormone present and active also in mammals, where it stimulates tissue glucose uptake. In the absence of insulin, ABA stimulates GLUT4 expression and glucose uptake in preadipocytes, quantitatively similarly to insulin^3,8^. The effect of ABA on lipid synthesis and storage in preadipocytes is, however, different from that of insulin: ABA does not induce preadipocyte differentiation and accumulation of triglycerides, instead promoting browning features, such as increased O_2_ consumption, mitochondrial content and expression of browning genes in white adipocytes *in vitro* and increased PGC-1α and UCP-1 expression and glucose uptake in the BAT of ABA-treated mice^8^.

In this study, experiments performed with two different techniques *in vitro* on L6 myoblasts (Fig. 1a) and *ex vivo* on murine skeletal muscle (Fig. 1c) demonstrate that ABA stimulates muscle glucose transport in the absence of insulin, and that activation of AMPK is responsible for this effect, as it is abrogated by inhibition of AMPK with dorsomorphin. ABA indeed increases phosphorylation of AMPK on Thr172 in L6 cells and in mouse muscle (Fig. 2b,d), and also stimulates AMPK transcription, as observed both by Western blot (Fig. 2b) and by RT-PCR (Fig. 2e).

The activation by ABA of AMPK, is in agreement with the metabolic effects exerted by ABA in skeletal muscle (this study) and in adipose tissue (AT)^8^. Indeed, AMPK directly phosphorylates and activates PGC-1α in skeletal muscle, and activation of PGC-1α increases GLUT4 expression^12^ and glycogen accumulation^13^. AMPK also activates PGC-1α in white visceral AT^17^, and PGC-1α increases expression of thermogenic and mitochondrial genes in human and murine adipocytes^18,19^. The transcription of PGC-1α is indeed upregulated by low-dose ABA in AT, both *in vitro* and *in vivo*^8^. In addition, AMPK is known to phosphorylate and inhibit the transcriptional activity of PPAR-γ, the chief regulator of adipogenesis, thereby inhibiting preadipocyte differentiation^20^ and accumulation of triglycerides^21^. AMPK-mediated suppression of PPAR-γ transcriptional activity has been linked to the anti-obesity effect of several natural products^20,22^. In line with an AMPK-dependent inhibition of adipogenesis and triglycerides accumulation in AT, chronic low-dose ABA significantly reduces BW in high-glucose fed mice and in humans^23^. AMPK is also an upstream positive regulator of p38 MAPK^24^, which was demonstrated to promote PPAR-γ phosphorylation on Ser122, thus preventing PPAR-γ mediated inhibition of GLUT4 expression^25^. Heterozygous PPAR-γ deficient mice have an improved insulin sensitivity and a reduced tendency to obesity^26,27^ and mice chimeric for wild-type and PPAR-γ null cells exhibit little or no contribution to adipose tissue formation by null cells^28^.

Natural products capable of activating AMPK, such as curcumin, genistein and ursolic acid are being proposed as treatments against obesity and the metabolic syndrome^20^. Differently from these natural compounds, ABA is an endogenous mammalian hormone^29^, the plasma levels of which increase after a glucose load in healthy subjects, but not in diabetic patients^9^: this fact allows to hypothesize that low endogenous plasma ABA may concur to the metabolic derangement of the metabolic syndrome (obesity, T2D and hyperlipidemia).

The role of Akt in the ABA signaling pathway is more difficult to dissect. In serum-starved L6 myoblasts, ABA induces an approximate 2-fold increase in both pAkt (Ser473) and total Akt, in the absence (Fig. 2a), as well as in the presence of 5 mM glucose (not shown). Preincubation of L6 myoblasts with AZD5363, a pan-Akt competitive inhibitor^30^, significantly increased pAMPK levels in ABA-treated compared with untreated cells (1.74 ± 0.34 *vs*. 1.41 ± 0.14, n = 5, p = 0.02), indicating presence of a moderating effect by Akt on ABA-induced AMPK activation. Indeed, activation of Akt by phosphorylation on both Thr308 and Ser473 inhibits AMPK phosphorylation on Thr172 by LKB1^31^. Akt lies at the crossroads between starved and fed state: in the fed state, insulin and high glucose favor the double phosphorylation of Akt on Ser473 and on Thr308^32,33^. It is possible that, despite the 12 hours serum starvation of L6 cells, a certain amount of Akt phosphorylated on Thr308 was present, accounting for the observed increase in pAMPK levels in ABA-treated L6 in the presence of AZD5363.

**Figure 6:**
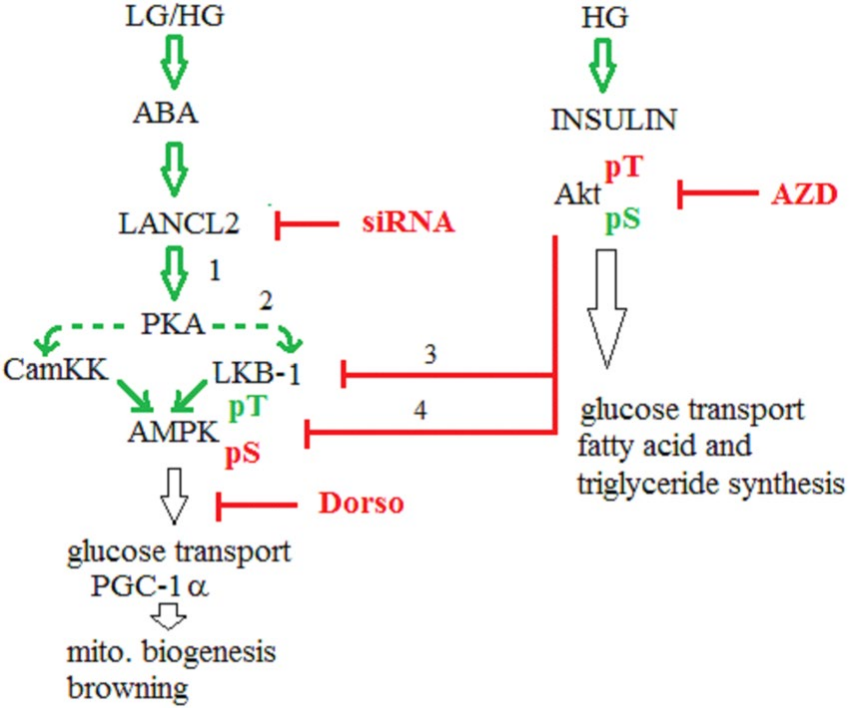
Hypothesis on the non-overlapping roles of ABA and insulin in muscle glucose transport and in adipocyte metabolism. Under conditions of high blood glucose (HG) insulin promotes phosphorylation of Akt on both Ser473 and Thr308^54^. Phosphorylation at both sites results in maximal activation^55^. Fully phosphorylated Akt prevents activation of AMPK by inhibition of LKB-1^31^ and by phosphorylation of AMPK on Ser485/491^56^. ABA, via LANCL2, promotes phosphorylation of AMPK on Thr172, activating the kinase both under conditions of low (LG) and of high glucose (HG). Activation of AMPK downstream of LANCL2 may occur *via* PKA-mediated phosphorylation of either or both kinases known to activate AMPK, i.e. LKB-1 and CamKK-β (dashed lines). (**1**) Activation of PKA downstream of the ABA/LANCL2 signaling pathway occurs in several human and rodent cell types^4,57,58^. (**2**) PKA can phosphorylate and activate LKB-1^39,40^ and can also phosphorylate CD38, leading to overproduction of the Ca^2+^-mobilizing second messenger cADPR^4^: this in turn could lead to an increase of the intracellular Ca^2+^ concentration, activating Ca^2+^-dependent CamKK. Insulin-activated Akt inhibits AMPK through several mechanisms: (**3**) - indirectly, by nuclear sequestration of LKB-1^31^ and, (**4**)- directly, by the hierarchical phosphorylation of Ser485, which prevents subsequent phosphorylation on Thr172 by LKB-1^33,56,59^. Downregulation of AMPK is necessary to allow an optimal anabolic response to high nutrient availability, and the opposite is also true: the ability of insulin and IGF-1 to stimulate protein synthesis is suppressed by AMPK. AMPK is an upstream activator of PGC-1α which increases GLUT4 expression and glycogen accumulation in skeletal muscle^12,13^ and is a master regulator of mitochondrial biogenesis^60^. Indeed, ABA stimulates PGC-1α transcription, mitochondrial production and O_2_ consumption in preadipocytes^8^. Downstream of the signaling pathway of insulin, Akt phosphorylated both on Ser473 and on Thr308 stimulates fatty acid and triglyceride synthesis in the white adipose tissue. Conversely, downstream of the signaling pathway of ABA, mitochondrial biogenesis and expression of browning genes in white adipocyte and brown adipose tissue glucose uptake are increased^8^. Dorso, dorsomorphin. AZD, AZD 5363. siRNA, LANCL2-specific siRNA.

Based on these results, a non-overlapping role for ABA and insulin in muscle glucose transport and in adipocyte metabolism can be envisaged (Fig. 6). Insulin induces phosphorylation of Akt on both Ser473 and Thr308, thus inhibiting AMPK and shifting the metabolic program from the starved to the fed state, activating not only glucose transport and metabolism, but also lipid and protein synthesis and adipogenesis. ABA instead induces phosphorylation of AMPK on Thr172, thereby activating the metabolic response to starvation and/or low glucose availability. This response includes stimulation of glucose transport in adipocytes, but not lipid synthesis, instead favoring UCP-1 expression, O_2_ consumption and mitochondrial biogenesis^8^.

The non-overlapping signaling pathways and metabolic effects of ABA and insulin in the response of muscle and adipose tissue to glucose allow to hypothesize a role for ABA as the first hormonal response, capable of stimulating muscle and adipocyte glucose uptake and oxidative metabolism in the presence of low glucose availability; persistence of hyperglycemia, indicating glucose availability in excess of normal tissue requirements, then triggers insulin release, which is however moderated by the concomitant action of ABA. Eventually, fully phosphorylated Akt downstream of the insulin signaling pathway in turn moderates activation by ABA of AMPK and starts the metabolic response to hyperglycemia, which includes lipid synthesis and storage. A different role for Akt phosphorylated on Ser473 alone, or both on Ser473 and on Thr308 might be envisaged also based on the facts that defective Ser473 phosphorylation affects only a subset of Akt targets^34^ and that different phosphatases specifically dephosphorylate these residues^35^. Phosphorylation of both Akt and AMPK at Ser473 and Thr172, respectively, with stimulation of muscle glucose uptake has been reported for other natural products^36^: thus, it is tempting to speculate that ABA may be the endogenous hormone responsible for activation of this pathway, the details of which remain to be clarified.

The ABA receptor LANCL2 appears to be involved in the ABA-induced effects on skeletal muscle, as silencing of LANCL2 significantly reduced ABA-stimulated glucose uptake (Fig. 1a,b) and AMPK phosphorylation (Fig. 2b) and PGC-1α expression (Fig. 2c) in L6 cells. The fact that the effect of ABA was not abrogated by LANCL2 silencing, despite the very high percentage of reduction of protein and mRNA expression (insets to Fig. 1a,b), suggests that other ABA receptors might be partly involved in the effect of ABA on muscle. LANCL1, a LANCL2 homolog, is indeed expressed in murine skeletal muscle at levels similar to those of LANCL2. Thus, it is possible that stimulation by ABA of AMPK phosphorylation and of PGC-1α expression, which retain a significant increase over control values in LANCL2-silenced cells (Fig. 1a,b), may depend also on LANCL1.

The kinase(s) activated by ABA and responsible for AMPK phosphorylation remain to be identified. Two kinases, LKB1 and Ca^2+^/calmodulin dependent protein kinase-β (CamKK-β) are known to phosphorylate and activate AMPK^37^. Activation of CamKK-β downstream of LANCL2 could be expected on the basis of previous findings in inflammatory cells, showing PKA-dependent phosphorylation and activation of the ADP-ribosyl cyclase CD38, resulting in an increase of the Ca^2+^-mobilizing second messenger cyclic ADP-ribose^4,38^. LKB-1 is also a target of PKA^39,40^. Thus, activation of PKA by LANCL2 could lead to the activation of both CamKK and LKB-1 (Fig. 6).

With dynamic micro-PET, we addressed one of the questions raised by a previous study in rats and in humans, i.e. which tissues are mainly responsible for the faster blood glucose clearance induced by low-dose oral ABA^7^. Glucose uptake in skeletal muscle increases 2-fold in ABA-treated compared with untreated rats during an oral glucose load (Fig. 3f). Given the high percentage of BW represented by skeletal muscle in rats, approximately 45%^41^, this increase of muscle glucose uptake may account for the accelerated blood glucose clearance observed in the ABA-treated compared with the control rats (Fig. 3d). BAT glucose uptake also increased approximately 2-fold in the ABA-treated rats compared with untreated controls, in line with previous observations^8^. BAT glucose uptake is a measure of its metabolic activity, which allows energy dissipation through heat production; thus, an increased BAT glucose uptake in the ABA-treated rats should increase the overall body energy expenditure. Indeed, male mice fed for 4 weeks a high-glucose diet containing ABA at the same low dose used in the rat OGTT experiments (1 µg/Kg BW) showed a slight reduction of BW compared with ABA-untreated controls (38.8 ± 0.93 vs. 40.2 ± 1.6). This trend towards a weight reduction of ABA-treated mice was confirmed when a 4-month ABA supplementation to a high-glucose diet was performed^23^.

To compare the physical performance in ABA-treated and –untreated mice, we adopted the running wheel test, which is often used to assess levels of general physical activity, as it allows a quantifiable measure of physical activity^42^. Open-field activity is instead mostly used to evaluate stress, anxiety, or locomotor response to novelty rather than overall spontaneous activity levels^43^. The mice were allowed access to the running wheel for a relatively short time (12 hours), during the night-time only and without prior acclimation, to reduce possible confounding aspects related to wheel running, such as alteration of behavior, metabolism and energy expenditure due to protracted exercise on the wheel^42^. Interaction with the wheel may depend in part on the curiosity for a new gadget; however, the facts that the ABA-treated animals kept the wheel running for approximately twice the time of their untreated peers, covered approximately twice their total distance (Fig. 4b) and reached a three-times higher maximal speed on the wheel, altogether indicate a significantly higher endurance of the ABA-treated animals, in line with the higher glycogen content detected in their muscles (Fig. 4e,f). The increased muscle glucose uptake *per se* could be responsible for an increased glycogen synthesis, as muscle glycogen synthase is allosterically activated by glucose 6-phosphate^44^. Genetic or pharmacological interventions that increase glycogen synthesis relative to glycogenolysis can indeed promote glucose tolerance^45,46^, and the cellular physiology controlling the flux through these opposite pathways is being explored for new anti-diabetic drug targets.

The fact that similar effects of chronic low-dose ABA as observed on CD1 mice fed a high-glucose diet, i.e. improvement of the glycemia profile during OGTT and increased muscle glycogen content, were also observed on TRPM2 KO mice, which are defective in insulin secretion, indeed suggests that ABA may at least in part substitute for insulin action on glycemia control and muscle glucose uptake. To address the issue of how the results obtained on a mouse model of impaired insulin secretion may translate to diabetic humans, future studies should investigate muscle glucose uptake in subjects with prediabetes or T2D, treated or not with microgram amounts of oral ABA.

Altogether, these results indicate that ABA shares with insulin the ability to stimulate muscle glucose uptake, albeit through a different signaling pathway, involving activation of AMPK. Interventions aimed at increasing AMPK activity are among the most successful current strategies to improve glucose tolerance in diabetic subjects. Physical activity and the AMPK activator metformin are currently prescribed as means to improve glucose tolerance in diabetic or prediabetic subjects, both acting through activation of AMPK. Results presented here suggest that ABA is the endogenous hormone in charge of AMPK activation and imply that low-dose oral ABA may alleviate insulin deficiency, as occurs in hypoinsulinemic TRPM2 KO mice, or contribute to reduce the dose of insulin required to control hyperglycemia in insulin-resistant T2D and in insulin-deficient T1D.

## Materials and methods

### siRNA-mediated silencing of LANCL2

Transient transfection of L6 cells was performed using the Nucleofector System (Amaxa GmbH, Cologne, Germany). L6 (2 × 10^6^ cells) were transfected using the Nucleofector program X-005 and R Kit with 2 µM rat LANCL2-targeting Stealth duplex short interference RNA (siRNA-L2, NM_001014187_stealth_208) or with 2 µM Stealth Negative Scramble Control (siRNA-SCR, NM_001014187_stealth_control_208) obtained from Invitrogen (Milan, Italy). After transfection, cells were resuspended in 4.5 mL of DMEM supplemented with FBS (10%) and incubated in a 5% CO_2_ at 37 °C. After 24 hours, the medium was replaced with serum-free DMEM and all experiments were performed 48 hours after transfection.

### Animals

Male Wistar rats (7-week old) and CD1 mice (6-week old) were purchased from Charles River (Milan, Italy). TRPM2 knock-out (TRPM2-KO) mice (on a C57BL/6 background) and their wild-type (WT) siblings, originally developed by Yamamoto *et al*.^47^, were obtained from Prof. A. Nencioni (IRCCS Ospedale Policlinico San Martino, Genova, Italy). Animals were housed at the animal facility of the same IRCCS Ospedale Policlinico San Martino. The protocol of animal use was approved by the Ethics Committee of the animal facility and all procedures involving animals were performed according to European Community directive 2010/63/EU.

### Glucose transport assays

#### *In vitro* 2-NBDG assay

L6 rat myoblasts were cultured overnight at 2 × 10^4^/well in a 96-well plate in DMEM (5 mM glucose) without serum. Cells were washed twice with KRH, pre-treated or not for 30 min at 37 °C with the AMPK inhibitor dorsomorphin (1 µM)^48^, and then incubated for 30 min at 37 °C with or without 100 nM ABA. The fluorescently labeled deoxyglucose analog 2-NBDG (50 µM) was added to each well and after 10 min the supernatant was removed, wells were washed once with ice-cold KRH, 50 µL KRH was added to each well and the mean fluorescence (λex 465 nm, λem 540 nm) of 10 acquisitions/well was calculated. Each experimental condition was assayed in at least 8 wells. Unspecific 2-NBDG uptake, determined in the presence of the glucose transport inhibitors cytochalasin B (20 μM) and phloretin (200 μM)^49^, was subtracted from each experimental value.

#### FDG uptake *in vitro* and *ex vivo*

L6 rat myoblasts (1 × 10^6^ cells) were cultured overnight in a 90 mm Petri dish in DMEM (5 mM glucose) without serum. The day after cells were incubated with or without ABA (100 nM final concentration) for 1 hour and then covered with 2 mL solution collected from an input vial containing 3 mL of DMEM medium (5 mM glucose) containing FDG at the concentration of 1 MBq/mL. Time-activity curves (TAC) of tracer uptake were then plotted using the Ligand Tracer White device (Ridgeview, Uppsala, Se)^50,51^. Briefly, this instrument consists of a beta-emission detector and a rotating platform harbouring a standard Petri dish. The rotation axis is inclined at 30° from the vertical, so that the medium covers the dish at nadir while the detector points at its zenith. All experiments consisted of 25 periodic rotations lasting one minute and divided into 4 intervals: (*a*) 25 sec in the system nadir (tissue fully immersed in the incubation medium); (*b*) 5 sec. 180° counter-clockwise rotation; (*c*) 25 sec under the detector at the system zenith and, finally, (*d*) 5 sec. 180° counter-clockwise rotation for cycle restart. At each cycle, the detector measures decay-corrected background and target counting rates (in counts per second, CPS) in phases *a* and *c*, respectively. FDG muscle TAC was thus obtained by subtracting background counting rate from the corresponding target value^52^. At the end of the experiment, an aliquot of 0.5 mL was sampled both from the input vial and from the Petri dish (output) to measure FDG concentration (MBq/ml) and thus tracer uptake for muscle unit mass^53^. FDG uptake was measured in the presence of cytochalasin B (20 μM) and phloretin (200 μM), to determine the background radioactivity.

TAC of FDG uptake were obtained also from murine muscle; briefly a small (~100 mg) sample was taken immediately after sacrifice from the quadriceps muscle of CD1 mice, starved for 12 hours. The tissue was stuck to the border of a Petri dish with octyl-cyanoacrylate (Dermabond, Ethicon, US) and covered with DMEM containing 12.5 mM glucose, containing, or not, 1 µM dorsomorphin. After 30 min incubation, FDG (1 MBq/ml), containing or not 100 nM ABA, was added and the analysis was started.

### High-glucose diet

6-week old male CD1 mice, or C57BL/6 mice genetically deficient for TRPM2 (TRPM2-KO) and their wild-type siblings (TRPM2-WT) were fed a standard chow and were administered glucose with the drinking water without (controls), or with the addition of synthetic ABA for 5 weeks. The concentrations of glucose and of ABA in the water were calculated considering the average daily volume drank by each mouse so as to reach a daily dose of 1 g/Kg BW of glucose and 1 μg/Kg BW of ABA. Food and water consumption were monitored 3 times per week: no significant difference between ABA-treated and -untreated animals was observed regarding food and water intake.

### Muscle performance

Male CD1 mice were fed a high-glucose diet with or without ABA (see above). At the end of the 4^th^ week, a wheel connected to a digital speedometer was positioned in each cage and the following parameters were measured for 12 hours: (i) the total distance traveled on the wheel, and, (ii) the time of rotation of the wheel. The tests were performed during the night time on animals with freely available food and water containing glucose alone (controls) or glucose with ABA.

### Muscle glycogen

Male CD1 mice, male TRPM2-WT and TRPM2-KO (6 per group) mice were fed a high-glucose diet, with or without ABA, as described above. At the end of the 5^th^ week, mice were sacrificed after a 4 hours fast and the medial thigh muscles (the same area investigated by micro-PET analysis) and the tibialis muscle were taken from each animal and immediately frozen in liquid nitrogen. Tissue samples were then weighed, homogenized in a specific buffer of the Glycogen Assay Kit (Cayman Chemical, Ann Arbor, MI) and the glycogen content was measured according to the same kit manufacturer’s instructions.

### Statistical analysis

Normal distribution of datasets was tested with the Vassarstats website for statistical computation (www.vassarstats.net). Continuous variables are presented as means ± SD. Comparisons were drawn by unpaired, two-tailed Student’s *t* test, if not otherwise indicated. Statistical significance was set at *p* < 0.05.

Materials, Western blot, shRNA lentiviral transduction, Real Time-PCR, Oral glucose tolerance tests, Experimental micro-PET scanning protocol see Supplementary Information.

## Supplementary information

Supplementary Information.
