# Supplementary material for: Insulin-independent stimulation of skeletal muscle glucose uptake by low-dose abscisic acid via AMPK activation
Source: Sci Rep. 2020 Jan 29;10:1454. doi: 10.1038/s41598-020-58206-0 (PMC6989460; doi:10.1038/s41598-020-58206-0)
Supplement: Supplementary file 1 — Supplementary Information. [file 41598_2020_58206_MOESM1_ESM.pdf]

Insulin-independent stimulation of skeletal muscle glucose uptake by low-dose abscisic acid via AMPK activation.

Mirko Magnone, Laura Emionite, Lucrezia Guida, Tiziana Vigliarolo, Laura Sturla, Sonia Spinelli, Ambra Buschiazzo, Cecilia Marini, Gianmario Sambuceti, Antonio De Flora, Anna Maria Orengo, Vanessa Cossu, Sara Ferrando, Ottavia Barbieri and Elena Zocchi.

SUPPLEMENTARY MATERIALS AND METHODS

Materials.

(±)-2-cis, 4-trans abscisic acid (ABA), the protease inhibitor cocktail for mammalian cells and AZD5362 were from Sigma (Milan, Italy). 2-NBDG and dorsomorphin were from Cayman (Milan, Italy). Anti-AMPK, anti-phospho AMPK (Thr172), anti-Akt and anti-phospho Akt (Ser473) antibodies were from EuroClone (Milan, Italy). Anti-LANCL2 antibody was obtained as described in [57]. FDG was produced at the Nuclear Medicine facility of the IRCCS Ospedale Policlinico San Martino. L6 rat myoblasts were obtained from ATCC and maintained in DMEM medium, containing 10% fetal calf serum (FCS) at 37°C under a humidified, 4% CO_2 atmosphere.

Western blot.

L6 rat myoblasts ($0.5x10^6$/well) were seeded in 6-well plates in DMEM with 10% FBS. After cell adhesion, cells were washed, cultured overnight at 37°C in DMEM with 5 mM glucose and processed for Western blot and glucose uptake experiments. For Western blot, the supernatant was removed, cells were washed once in Krebs-Ringer HEPES buffer (KRH) and then incubated in KRH with 5 mM glucose for 60 min at 37°C without or with 100 nM ABA. The supernatant was removed and cells were scraped in 100 µL lysis buffer (20 mM Tris-HCl pH 7.4, 150 mM NaCl, 1 mM EDTA, 1% NP40) containing a protease inhibitor cocktail. After brief sonication, the protein concentration was determined on an aliquot of each lysate. Western Blot experiments were performed also on quadriceps samples freshly isolated from mice and incubated for 30 and 60 minutes with or without 100 nM ABA; after incubation, muscles were lysed with Tissue Lyser (Qiagen, Milan, Italy), centrifuged for 10 min at 12000 x g and the supernatants were analyzed by Western Blot. SDS-PAGE (on 10% gels) and protein transfer to a nitrocellulose membrane (Bio-Rad, Milan, Italy) were performed according to standard procedures. The membrane was blocked for 1 h with 20 mM Tris-HCl pH 7.4, 150 mM NaCl, 1% Tween 20 (TBST) containing 5% non-fat dry milk and incubated for 1 h at room temperature with the primary antibody (anti LANCL2, anti-phospho AMPK or anti-phospho Akt and anti-vinculin as reference protein). After washing with TBST, the membrane was incubated with an anti-rabbit IgG antibody conjugated with horse radish peroxidase (HRP) (Santa Cruz Biotechnology, Dallas, TX, USA) and developed with Immobilon™ Western Chemiluminescent HRP Substrate (Millipore, Milan, Italy). Band intensity was evaluated with the Chemidoc system (Bio-Rad). After

membrane stripping, a second incubation with an anti-AMPK or an anti-Akt primary antibody was performed and the incubation with the secondary HRP-conjugated antibody was repeated.

shRNA lentiviral transduction

To obtain L6 cells stably silenced LANCL2, the following protocol was used. The lentiviral plasmids encoding for a control shRNA (shRNA-SCR) and for rLANCL2-shRNA (shRNA-L2) were purchased from VectorBuilder (CA, United States). Lentiviral vector particles (LVPs) were generated in Lenti-HEK293 cells. Briefly, HEK 293T cells were plated ($7x10^5$ cells on 6-cm plates) in Dulbecco's modified Eagle medium, 5% fetal bovine serum, and 0.1% penicillin-streptomycin. After 24 hours, cells were cotransfected with a Δ891 and a VSV-G encoding vector along a shRNA transfer lentiviral plasmid (SCR-shRNA or L2-shRNA) using *TransIT*® Transfection Reagent (Mirus, Madison, USA). After 24 hours, the 293T medium was changed with Dulbecco's modified Eagle medium, 20% fetal bovine serum, and 10% penicillin-streptomycin to promote viral production. The supernatant containing lentiviral particles was collected 48 and 72 hours after transfection, filtered with a 0.45-μm-diameter filter, and used to infect L6 cells (1x106 cells) in the presence of protamine sulfate (final concentration 5 μg/mL). After the second cycle of infection, cells were selected with puromycin (5 μg/mL). The knockdown efficiency was validated by evaluating LANCL2 mRNA and protein levels by Real Time PCR and Western Blot analysis, respectively.

Real Time-PCR.

Total RNA was extracted from L6 cells using the RNeasy micro kit (Qiagen, Milan, Italy) and from muscle biopsies using Qiazol (Qiagen), both according to the manufacturer's instructions, and reverse transcribed into cDNA using the iScript™ cDNA Synthesis Kit (Bio-Rad). The cDNA was used as template for real-time PCR analysis: reactions were performed in an iQ5 real-time PCR detection system (Bio-Rad) following the experimental conditions described before [58]. The rat-specific primers were designed by using Beacon Designer 2.0 software (Bio-Rad) and their specific sequences for LANCL2, GAPDH and β-Actin were described before [58]. PCR specific primers for rat PGC-1α and mouse AMPK, were the following: rat PGC-1α, 5'-CTCTGCGGTATTCGTCCCTC -3' (forward) and 5'- GCACACATCGCAATTCTCCC-3' (reverse); mouse AMPK, 5'-AGAAGCAGAAGCACGACGG-3' (forward) and 5'-TTGCCCACCTTCACTTTCCC-3' (reverse). Specific primers for rat LANCL2, GAPDH and actin were described in [58]. Specific primers for mouse housekeeping genes, ubiquitin and β2-microglobulin were described in [9].

Oral glucose tolerance tests.

Rats (7/group) were fasted for 17 hours before the test. After mild sedation with diazepam, an oral glucose tolerance test (OGTT) was performed: 1 g/Kg body weight (BW) glucose, without (control) or with 1 μg/Kg BW of (±)-2-cis, 4-trans ABA (Sigma, Milan, Italy) (ABA), was administered by

gavage in approximately 300 µL water solution. After gavage, anesthesia was induced by intramuscular administration of ketamine (33 mg/Kg) and xylazine (5 mg/Kg) (Imalgere 1000, Milan, Italy and Bio98 Srl, Milan, Italy, respectively) and rats underwent a micro-PET analysis.

Mice were fasted for 17 hours before the OGTT, then 1 g/Kg BW glucose was administered by gavage in 150 µL water solution. Blood was drawn from the tail vein before gavage (time zero) and 15, 30, 60 and 120 min after gavage: glycemia was immediately measured with a glucometer, each measure being performed in duplicate. The area under the curve (AUC) of glycemia was calculated with the trapezoidal rule, from the blood glucose concentrations measured at the indicated time-points after gavage, relative to the value of glycemia at time 0 (before gavage).

Experimental micro-PET scanning protocol.

In vivo imaging was performed according to a published protocol [61] Daily quality controls always documented a radiochemical purity of in-house produced FDG ≥ 98%. Immediately after anesthesia following gavage (see above), the rats were positioned on the bed of a dedicated micro-PET system (Albira, Bruker, Billerica, MA, USA), centering the scanner field of view on the chest. A dose of 30-45MBq of FDG was injected through a tail vein exactly 15 min after gavage, and a list mode acquisition lasting 50 min was started. Blood was obtained from the tail vein before gavage (time zero), and 15 (immediately before FDG injection), 30 and 60 min after gavage: glycemia was immediately measured with a glucometer, and an aliquot of each blood sample anticoagulated with heparin was immediately centrifuged at 22,000 x *g* for 30 sec and plasma aliquots were stored at -20°C for the determination of insulinemia.

In a random sequence, each rat underwent an OGTT and an OGTT+ABA, one week apart.

The whole dataset was thus binned using the following framing rate: 10 x 15 sec, 5 x 30 sec, 2 x 150 sec, 6 x 300 sec, 1 x 600 sec. PET data were reconstructed using a maximal likelihood expectation maximization method (MLEM). An experienced observer, unaware of the experimental type of analyzed model, identified a volume of interest (VOI) in the left ventricular chamber. Then, the computer was asked to plot the time-concentration curve within this VOI throughout the whole acquisition to define tracer input function. Whole body FDG clearance (in µL x min^{-1}x g^{-1}) was calculated, using the conventional stochastic approach, as the ratio between injected dose and integral of input function from 0 to infinity, fitting the last 20 min with a mono-exponential function [62]. This value was multiplied by serum glucose level to measure whole body glucose consumption that was normalized for body weight and expressed as nmol x min^{-1}x g^{-1}.

Thereafter, all dynamic scans were processed according to the Gjedde-Patlak [63] graphical approach to compartmental analysis by using the routine of a dedicated software (PMOD, Zurich, Switzerland). Briefly, the software utilizes the input function and transforms the original tissue activity

measurements by fitting the data in each voxel with the slope of the regression line defined by the model. In all cases, lumped constant value was set at 1. Further VOIs were drawn on the obtained parametric maps to estimate the regional metabolic rate of glucose (MRGlu) in the medial thigh region, comprising the muscles biceps femoris, gracilis, semitendinosus and rectus femoris.

REFERENCES

[61] Marini, C., et al. Divergent targets of glycolysis and oxidative phosphorylation result in additive effects of metformin and starvation in colon and breast cancer. *Sci Rep* **22**:19569 (2016).

[62] Iozzo, P., et al. ^{18}F-FDG assessment of glucose disposal and production rates during fasting and insulin stimulation: a validation study. *J Nucl Med* **47**:1016-1022 (2006).

[63] Patlak, C.S., Blasberg, R.G., Fenstermacher, J.D. Graphical evaluation of blood-to-brain transfer constants from multiple-time uptake data. *J Cereb Blood Flow Metab* **3**:1-7 (1983).
